# Low-Glucose Metabolic Remodeling Drives Reversible Sequestration of Yeast S-Adenosylmethionine (AdoMet) Synthase

**DOI:** 10.3390/cells15141267

**Published:** 2026-07-14

**Authors:** Hasnat Ahmed, Ahmad Sleiman, Mohamad Rahal, Eyas Hanbali, Marwa Aboumourad, Daniel Adebayo, Eseiwi Obaseki, Lindsey Kreinbring, Scott Miller, Elias Ayoub, Allison Jacobson, Andrew Rassam, Xun Bao, Jing Li, Hanaa Hariri

**Affiliations:** 1 Biological Sciences Department, Wayne State University, Detroit, MI 48202, USA; 2 Department of Oncology, School of Medicine, Wayne State University, Detroit, MI 48201, USA; 3 Karmanos Cancer Institute, Detroit, MI 48201, USA

**Keywords:** S-adenosylmethionine (AdoMet), metabolic flux, nutrient stress, enzyme assemblies

## Abstract

S-adenosylmethionine (AdoMet) is a central metabolite required for methylation and sulfur metabolism, synthesized in *Saccharomyces cerevisiae* by the cytosolic enzymes Sam1 and Sam2. While transcriptional and metabolic regulation of AdoMet biosynthesis have been extensively studied, how metabolic state influences the spatial organization of AdoMet synthases remains incompletely understood. Herein, we show that Sam1 undergoes robust and reversible relocalization into cytosolic assemblies under low-glucose metabolic states. These assemblies are dynamic and dissolve rapidly upon glucose refeeding in a manner that requires metabolizable glucose, indicating regulation by metabolic flux. While Sam1 assemblies associate with stress granules during prolonged starvation, a substantial fraction form independently under acute low-glucose conditions, indicating that Sam1 relocalization is not fully coordinated with canonical stress granule formation. Untargeted metabolomic analysis indicates that AdoMet pools are broadly maintained, while a targeted fluorescence-based enzymatic assay detects a modest but reproducible reduction in AdoMet under low-glucose conditions. Together, these findings define a metabolically gated mechanism for reversible Sam1 sequestration and support a model in which spatial organization of metabolic enzymes is coupled to cellular metabolic state.

## 1. Introduction

S-adenosylmethionine (AdoMet) is a central metabolic intermediate that functions as a universal methyl donor for DNA, RNA, protein, and lipid methylation and serves as a precursor for polyamine synthesis and sulfur metabolism [1,2,3,4]. Through these roles, AdoMet links biosynthetic capacity, redox balance, and cellular growth [5,6]. In *Saccharomyces cerevisiae*, AdoMet is synthesized in the cytosol by the enzymes Sam1 and Sam2 [7,8,9]. While transcriptional and metabolic regulation of AdoMet biosynthesis have been characterized, far less is known about how AdoMet-producing enzymes like Sam1 and Sam2 are regulated at the post-translational or spatial level, particularly in response to changes in nutrient availability [5,10].

Early foundational work from Paul Srere and colleagues proposed that metabolic enzymes form dynamic, spatially organized cellular compartments to regulate metabolic pathways in space and time [11]. Increasing evidence suggests that metabolic regulation is not governed solely by enzyme abundance or catalytic activity, but also by their localization and spatial organization within the cell [12,13,14,15]. Multiple metabolic enzymes have been shown to reversibly assemble into cytosolic structures in response to nutrient availability and cellular stress [15,16,17,18,19]. These assemblies have been proposed to serve diverse functions, including storage of inactive enzymes, protection from degradation, or organization of metabolic pathways [20,21]. For example, enzymes involved in de novo purine biosynthesis can assemble into the “purinosome,” a dynamic multienzyme complex proposed to enhance pathway coordination under purine-depleted conditions [22]. Similarly, studies of the fatty acid biosynthetic pathway have shown that starvation induces reversible sequestration of fatty acid synthase Fas1 and may modulate metabolic flux into this pathway, potentially by restricting enzyme access to substrates or interaction partners [23]. Despite these observations, the molecular mechanisms and biological significance of spatial reorganization of metabolic enzymes remain poorly understood. Whether enzyme relocalization represents a regulated mechanism of metabolic control and the metabolic conditions under which it occurs remain areas of active investigation.

Although many starvation-induced metabolic enzyme assemblies have been reported to form independently of stress granules, recent work has increasingly linked metabolic state to stress granule biology. Stress granules are dynamic and reversible ribonucleoprotein assemblies that form in response to nutrient limitation and environmental stress and are thought to reorganize cellular metabolism and translation during stress adaptation [24,25,26]. In particular, a recent study demonstrated that the AdoMet metabolite regulates stress granule assembly and that Sam1 can be recruited to stress granules under prolonged starvation conditions [26]. Importantly, however, Sam1 is not recruited to stress granules during acute glucose deprivation despite robust stress granule formation, indicating that Sam1 relocalization is not a general feature of stress granule assembly but instead occurs in a stress- and metabolic state-dependent manner [26]. These findings suggest that distinct nutrient states may differentially regulate Sam1 assembly and its association with stress granules. However, the metabolic conditions that promote Sam1 relocalization, and the extent to which Sam1 assemblies are coordinated with stress granules, remain incompletely understood.

Herein, we investigate how metabolic state regulates the spatial organization of the yeast AdoMet synthases Sam1 and Sam2. We show that sustained low-glucose conditions drive robust formation of Sam1-containing assemblies. These structures are dynamic and reversible, dissolving rapidly upon glucose refeeding in a manner that requires metabolizable glucose, indicating regulation by metabolic flux. While Sam1 assemblies associate with stress granules during prolonged starvation in a growth-dependent manner, a substantial fraction form independently under acute low-glucose conditions (0.1% glucose), suggesting distinct assembly states. Finally, metabolomic analysis reveals that Sam1 relocalization occurs within a rewired metabolic state characterized by reduced central carbon metabolism and increased amino acid, sulfur, and redox pathways, without substantial depletion of bulk AdoMet levels. Together, our findings support a model in which spatial sequestration of Sam1 represents a regulated response to metabolic state, linking enzyme organization to cellular metabolic adaptation under nutrient limitation.

## 2. Materials and Methods

### 2.1. Yeast Strains, Media, and Growth Conditions

Yeast genetic manipulations were conducted using classical yeast knock-in/out protocols. All strains and genetic manipulations were verified by sequencing and phenotype. The prototrophic CEN.PK strain background was used in all experiments [4]. Gene deletions were carried out using homologous recombination [27]. The strains and primers used are listed in Tables S1 and S2. Yeast transformations were performed using the lithium acetate method. Strains were selected using antibiotics. All chemicals used to make the yeast media were purchased from Sigma-Aldrich St. Louis, MO, USA (succinic acid, sodium hydroxide, ammonium sulfate, yeast nitrogen base without amino acids, or ammonium sulfate). Yeast cells were grown in various media conditions: YPD 2% glucose, YPD 0.1% glucose, YPL 2%, and YP 2% glycerol. Yeast cultures were grown at 30 °C with shaking (180–200 rpm) until reaching the desired optical density (OD600 0.5–0.8) in the exponential growth phase.

### 2.2. Microscopy, Imaging, and Image Processing

Imaging of live yeast cultures was performed by using the EVOS M5000 Cell Imaging System (Thermo Fisher Scientific, Waltham, MA, USA) at room temperature. Yeast cultures were grown to mid-log phase, harvested, and washed in media with no dextrose before being transferred to conditional media. Before imaging, cells were pelleted (3000× *g* for 5 min at room temperature), washed, and resuspended in a small volume of media without a carbon source. Then, 3–5 µL of the dense yeast suspension was transferred to a glass slide for imaging. Foci formation was quantified in different media conditions and at the indicated timepoints. Image analysis was performed using Fiji (ImageJ 1.54r). The degree of colocalization between *SAM1* and *SAM2* was determined manually as the percentage of the number of GFP foci that overlapped with mCherry foci divided by the total number of GFP foci. Because the analysis involved discrete cytoplasmic puncta rather than diffuse fluorescence, colocalization was assessed by scoring puncta overlap in individual cells. Image analysis and quantification were performed in a blinded manner to the experimental conditions to minimize observer bias. Foci formation was also determined manually. Each experiment was performed at least three independent times, with a minimum of 50 cells counted per replicate (3 replicates per experiment). Data represent the mean ± SEM. Data were assessed for normality using the Shapiro–Wilk test prior to statistical analysis. Statistical significance between groups was assessed using an unpaired two-tailed Student’s *t*-test. A *p*-value < 0.05 was considered statistically significant.

### 2.3. Western Blot Analysis of Yeast Proteins

Yeast cells were grown in appropriate media to the desired optical density (e.g., OD600 0.5–0.8) and harvested via centrifugation at 3000× *g* for 5 min at 4 °C. The cell pellet was washed twice with ice-cold phosphate-buffered saline (PBS) and resuspended in lysis buffer (50 mM Tris-HCl, pH 7.5, 150 mM NaCl, 1% Triton X-100) supplemented with protease inhibitors (1 mM PMSF, 5 μg/mL leupeptin, 10 μg/mL pepstatin, 8 μg/mL aprotinin). Chemicals were purchased from Sigma-Aldrich. Cells were lysed by mechanical disruption using glass beads (0.5 mm diameter) in a bead-beater for 6 cycles of 1 min on and 1 min off at 4 °C. Cell lysates were clarified via centrifugation at 16,000× *g* for 15 min at 4 °C, and the supernatant containing soluble proteins was collected. Protein concentration was determined using the Bradford assay (Bio-Rad, Hercules, CA, USA). Equal amounts of protein (20–50 µg) were mixed with SDS loading buffer (final concentration: 62.5 mM Tris-HCl, pH 6.8, 10% glycerol, 2% SDS, 5% β-mercaptoethanol, 0.01% bromophenol blue) and boiled at 95 °C for 5 min. Proteins were separated by SDS-PAGE on a 10–12% polyacrylamide gel and transferred to a polyvinylidene difluoride (PVDF) membrane using a semi-dry or wet transfer system at 100 V for 1 h. The membrane was blocked with 5% non-fat dry milk in Tris-buffered saline with 0.1% Tween-20 (TBS-T) for 1 h at room temperature. The membrane was then incubated overnight at 4 °C with primary antibodies diluted in TBS-T containing 5% milk or bovine serum albumin (BSA). After three washes in TBS-T (5 min each), the membrane was incubated with the appropriate horseradish peroxidase (HRP)-conjugated secondary antibody diluted in TBS-T for 1 h at room temperature. The membrane was washed three additional times with TBS-T before detection. Protein bands were visualized using enhanced chemiluminescence (ECL) reagent and imaged with a chemiluminescence detection system.

### 2.4. AdoMet Enzyme Activity Assay

Yeast cells were cultivated in liquid YPD medium at 30 °C and grown to an OD A600 of 0.8–1.2, followed by passage into either YPD or YPGE medium with a starting A600 of 0.2 and grown at both 30 °C and 37 °C until an A600 of 0.8–1. Relative cellular SAM levels were determined using the SAM (AdoMet) Bridge-It fluorometric assay kit (Mediomics, St. Louis, MO, USA, #NC1099716) as per the manufacturer’s recommendations. Briefly, 1 OD A600 of yeast was pelleted/condition, and cellular AdoMet was extracted using the provided CM assay buffer by mechanical cell lysis with glass beads at ambient temperature for 1 h, and the supernatant was clarified by centrifugation (15,000× *g* for 15 min). The resulting supernatant (2 µL) and 18 µL of the provided AdoMet assay solution were mixed and incubated at ambient temperature for 30 min. Subsequent AdoMet-dependent fluorescence was determined using a SpectraMax (Molecular Devices, San Jose, CA, USA) microplate reader with excitation of 485 nm and emission of 665 nm. This experiment was repeated 3 times with *n* = 3–5 biological replicates per experiment. Statistical analyses were conducted on all replicates (*n* = 8) using the independent samples *t*-test. Normality was assessed with the Shapiro–Wilk test, which showed no significant deviation from a normal distribution (W(20) = 0.92, *p* = 0.123). A *p*-value of <0.05 was considered statistically significant.

### 2.5. Metabolite Analysis

Each yeast sample was collected in 80% ice-cold methanol and sonicated twice for 1 min in in an ice bath. The supernatant was transferred to a new 2 mL microcentrifuge tube, and 0.8 mL 80% methanol (cooled to −80 °C) was added to the precipitate, followed by vortex-mixing and centrifugation. Supernatant from 2 extractions was combined and dried in a CentriVap Refrigerated Centrifugal Concentrator (Labconco, Kansas City, MO, USA) at 10 °C. Samples were then analyzed using LC-MSMS following this published protocol [28]. Raw metabolite abundances were normalized to cell number (or OD) and log_2_-transformed prior to analysis. Principal component analysis (PCA) was performed on log_2_-transformed data to assess global differences between conditions, with 95% confidence ellipses shown. Differential analysis between 0.1% YPD and 2% YPD was conducted using Welch’s two-tailed *t*-test, and *p*-values were adjusted using the Benjamini–Hochberg false discovery rate (FDR). Metabolites were considered significant if FDR < 0.05 and |log_2_ fold change| > 1, and results were visualized using volcano plots. For heatmaps, data were row-scaled (z-score) and hierarchically clustered using Euclidean distance and complete linkage. For selected metabolites, bar plots display mean ± SD of log_2_-transformed values with individual replicates overlaid; statistical significance is indicated as *** *p* < 0.001, ** *p* < 0.01, and * *p* < 0.05, based on FDR-adjusted *p*-values.

## 3. Results

### 3.1. Low-Glucose Conditions Drive Sam1/Sam2 Assembly

Previous studies have reported that the yeast AdoMet synthases Sam1 and Sam2 can form cytosolic foci during prolonged growth and the stationary phase [26,29], but the environmental triggers and dynamics of this relocalization remain incompletely defined. We therefore sought to systematically examine Sam1 localization across defined nutrient conditions.

**Figure 1:**
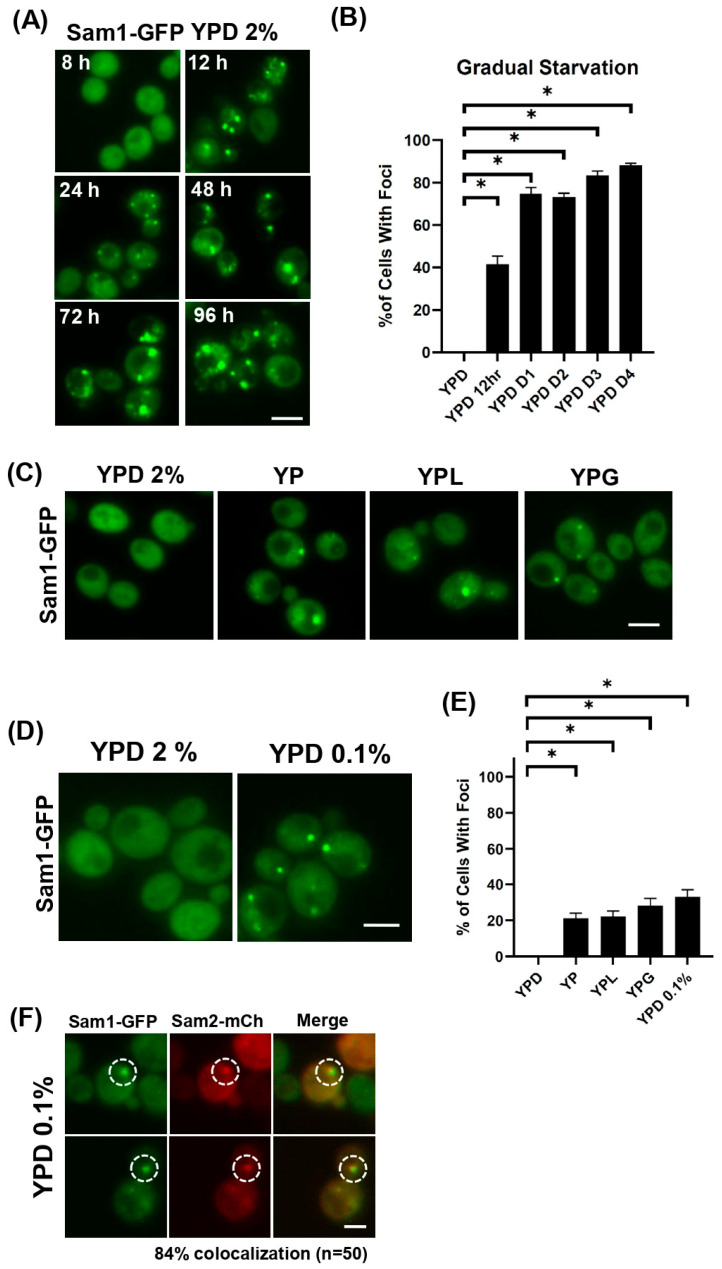
Sam1 accumulates into foci upon glucose starvation and colocalizes with Sam2. (**A**) Time course of Sam1-GFP relocalization during progressive nutrient depletion. Cells were grown to log phase in YPD (2% glucose) and subjected to starvation for the indicated times (8–96 h). Cells were imaged at 8, 12, 24, 48, 72, and 96 h of growth. Representative images of cells with foci are shown. Foci begin to appear at 12 h and increase in number with longer starvation. (**B**) Quantification of the percentage of cells containing Sam1-GFP foci under gradual starvation conditions shown in (**A**). Data represent mean ± SD from *n* = 3 independent experiments (*n* = 50 cells per condition). Statistical significance between groups was assessed using an unpaired two-tailed Student’s *t*-test. A *p*-value < 0.05 was considered statistically significant. Asterisks (*) denote significant differences as indicated in the figure. (**C**) Sam1-GFP localization in cells starved for 8 h under the indicated conditions: YPD (2% glucose), YP (no glucose), YPL (lactate), YPG (glycerol), and YPD 0.1% (glucose limitation). Foci formation is observed in all conditions except YPD 2%. (**D**) Sam1-GFP localization in cells grown in YPD (2% glucose) or subjected to low-glucose conditions (YPD 0.1%) for 8 h. Foci are observed under reduced glucose conditions but not in standard YPD. (**E**) Quantification of the percentage of cells containing Sam1-GFP foci under the indicated conditions in (**C**,**D**). Data represent mean ± SD from *n* = 3 independent experiments (*n* = 50 cells per condition). Statistical significance between groups was assessed using an unpaired two-tailed Student’s *t*-test. A *p*-value < 0.05 was considered statistically significant. Asterisks (*) denote significant differences as indicated in the figure. (**F**) Colocalization of Sam1-GFP and Sam2-mCherry (dashed circles) under glucose-limited conditions (YPD 0.1% for 8 h). Representative merged images show overlapping cytosolic foci. Single-plane images are shown. Degree of colocalization was determined manually as the percentage of the number of GFP foci that overlapped with mCherry foci divided by the total number of GFP foci. Scale bars: 2 μm.

To do this, first, we monitored endogenously tagged Sam1-GFP during progressive nutrient depletion as cells transitioned from log phase into stationary phase. Consistent with previous observations, Sam1-GFP formed cytosolic foci gradually over time, with an increasing fraction of cells exhibiting foci between 8 and 96 h of growth (Figure 1A,B). At later time points, the majority of cells contained Sam1-positive foci, indicating that relocalization is a progressive response to sustained nutrient limitation.

Because extracellular pH decreases during gradual starvation as cells grow into stationary phase, we next asked whether pH changes alone are sufficient to drive Sam1 relocalization. To test this, we examined Sam1-GFP localization under controlled pH conditions in the presence of glucose. We found that Sam1-GFP remained largely diffuse and did not form robust cytosolic foci, indicating that pH changes alone are not sufficient to account for Sam1 relocalization during gradual nutrient depletion (Figure S1A).

To determine whether Sam1 relocalization represents a general response to stress or is specific to metabolic conditions, we examined Sam1-GFP localization under heat (37 °C) and cold shock (ice bath) and found that Sam1-GFP retained largely diffuse localization under these conditions and did not form robust cytosolic foci (Figure S1B).

We next sought to define more precisely the nutrient conditions that trigger Sam1 relocalization. To this end, we examined Sam1-GFP localization in cells grown in rich glucose media (YPD, 2% glucose), glucose-free media (YP; 0% glucose), and media containing non-fermentable carbon sources (2% lactate, YPL; 2% glycerol, YPG), as well as under glucose-limited conditions (YPD 0.1%). As expected under glucose-replete conditions (YPD), Sam1-GFP was diffusely localized throughout the cytosol (Figure 1C). Growth in alternative carbon sources or glucose-free media resulted in an increase in the fraction of cells containing Sam1-GFP foci (Figure 1C). Notably, however, reduced glucose concentration (YPD, 0.1% glucose) induced robust formation of Sam1-GFP foci (Figure 1D,E). Together, these findings indicate that Sam1 relocalization occurs under conditions of sustained metabolic limitation.

Because yeast expresses a second AdoMet synthase, Sam2, we next asked whether the two paralogs localize to the same structures (Figure 1F and Figure S1C). Imaging of endogenously tagged Sam1-GFP and Sam2-mCherry revealed extensive colocalization (84%) within cytosolic foci under glucose-limited conditions (YPD, 0.1% glucose), indicating that Sam1 and Sam2 assemble into shared structures (Figure 1F and Figure S1C). Finally, we tested whether Sam1 relocalization depends on the presence of Sam2. We found that, in *sam2*Δ cells, Sam1-GFP foci formation was unchanged (Figure S1D), indicating that Sam1 relocalization is an intrinsic response and does not require Sam2.

Together, these findings demonstrate that Sam1 undergoes condition-specific relocalization into cytosolic foci under defined metabolic states, with robust induction under glucose-limited conditions (YPD, 0.1% glucose), establishing a framework to examine the nature and functional context of these assemblies.

### 3.2. Sam1 Assemblies Associate with Stress Granules Under Sustained Metabolic Stress

Previous work demonstrated that Sam1 can associate with stress granules during prolonged growth into stationary phase, where its recruitment follows stress granule assembly and depends on cellular metabolic state [26]. We therefore asked whether the Sam1 foci observed under our conditions also correspond to stress granule-associated structures.

**Figure 2:**
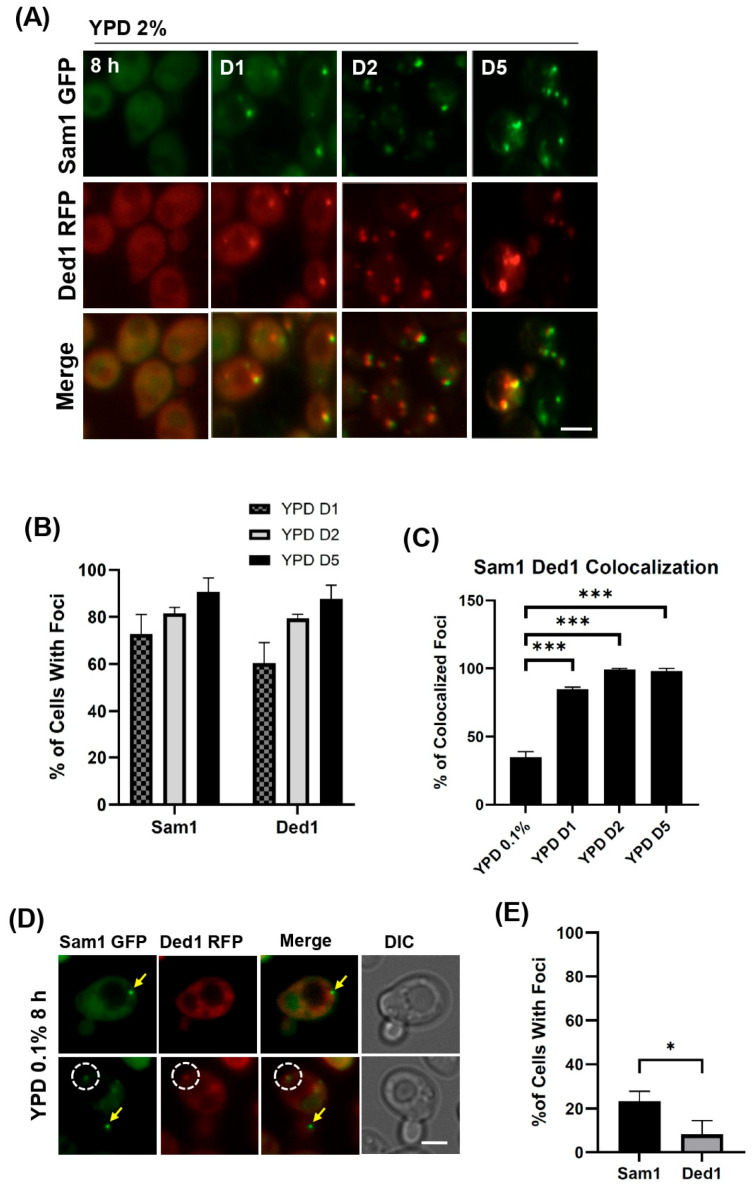
Sam1 foci colocalize with stress granules during prolonged glucose starvation. (**A**) Representative fluorescence images of cells co-expressing Sam1-GFP and Ded1-RFP under glucose-limited conditions (YPD 0.1%) and during progressive starvation (day 1, day 2, and day 5). Cells were grown to log phase in YPD (2% glucose) for the indicated durations (8 h, 1 d, 2 d, and 5 d). Sam1-GFP forms foci that increasingly colocalize with Ded1-marked stress granules over time. Merged images show colocalization of overlapping foci. (**B**) Quantification of the percentage of cells containing Sam1-GFP or Ded1-RFP foci under prolonged starvation conditions (YPD D1, D2, and D5). Data represent the proportion of total cells containing foci for each protein at each time point (*n* = 3 independent experiments (*n* = 50 cells per condition)). (**C**) Quantification of the percentage of Sam1-GFP foci that colocalize with Ded1-RFP foci across starvation conditions (YPD 8 h, D1, D2, D5, and YPD 0.1% 8 h). *n* = 3 independent experiments (*n* = 50 cells per condition). Reduced colocalization is observed under short-term low-glucose conditions (YPD 0.1% 8 h) compared to prolonged starvation. Statistical significance between groups was assessed using an unpaired two-tailed Student’s *t*-test. A *p*-value < 0.05 was considered statistically significant (*** *p* < 0.001). (**D**) Representative images of Sam1-GFP and Ded1-RFP localization in cells (dashed circles) subjected to low-glucose conditions (YPD 0.1%) for 8 h. Yellow arrows indicate limited colocalization between Sam1 and Ded1 foci under these conditions. (**E**) Quantification of the percentage of cells containing Sam1-GFP or Ded1-RFP foci in YPD 0.1% for 8 h, showing differences in foci formation between the two proteins (*n* = 3 independent experiments, *n* = 100 cells), Student’s *t*-test * *p* < 0.05. Scale bars: 2 μm.

To address this, we co-imaged Sam1-GFP with the stress granule marker Ded1-RFP during progressive and acute glucose depletion. Consistent with prior observations, both Sam1 and Ded1 formed cytosolic foci over time as cells grew into stationary phase, and the majority of Sam1 assemblies colocalized with Ded1-positive puncta at later time points (Figure 2A–C). These results indicate that under prolonged nutrient limitation, Sam1 is efficiently recruited to stress granules.

We next examined Sam1-GFP and Ded1-RFP localization under acute glucose-limited conditions (YPD, 0.1% glucose), where Sam1 foci formation is strongly induced (Figure 1D,E). Interestingly, under these conditions, Sam1-positive foci were more abundant than Ded1-positive puncta (Figure 2D,E). Quantitative analysis revealed that only a subset (~30%) of Sam1-GFP foci colocalized with Ded1-RFP in YPD 0.1% glucose (Figure 2C). Thus, while Sam1 retains the ability to associate with stress granules during gradual starvation, a substantial fraction of Sam1 assemblies do not associate with Ded1-positive structures under acute glucose-limited conditions.

Together, these findings indicate that Sam1 relocalization is partially coupled to stress granule formation, but that this association is strongly condition dependent. Under prolonged gradual nutrient depletion, Sam1 is efficiently recruited to stress granules, whereas under acute glucose-limited conditions (YPD, 0.1% glucose), Sam1 forms additional assemblies that may be distinct from, or only transiently associated with, canonical stress granules.

### 3.3. Sam1 Assemblies Are Dynamic and Dissolve in a Metabolism-Dependent Manner

Stress-induced assemblies can represent either dynamic, regulated structures or static aggregates. To distinguish between these possibilities, we next asked whether Sam1 foci are reversible and whether their dissolution depends on metabolic activity.

**Figure 3:**
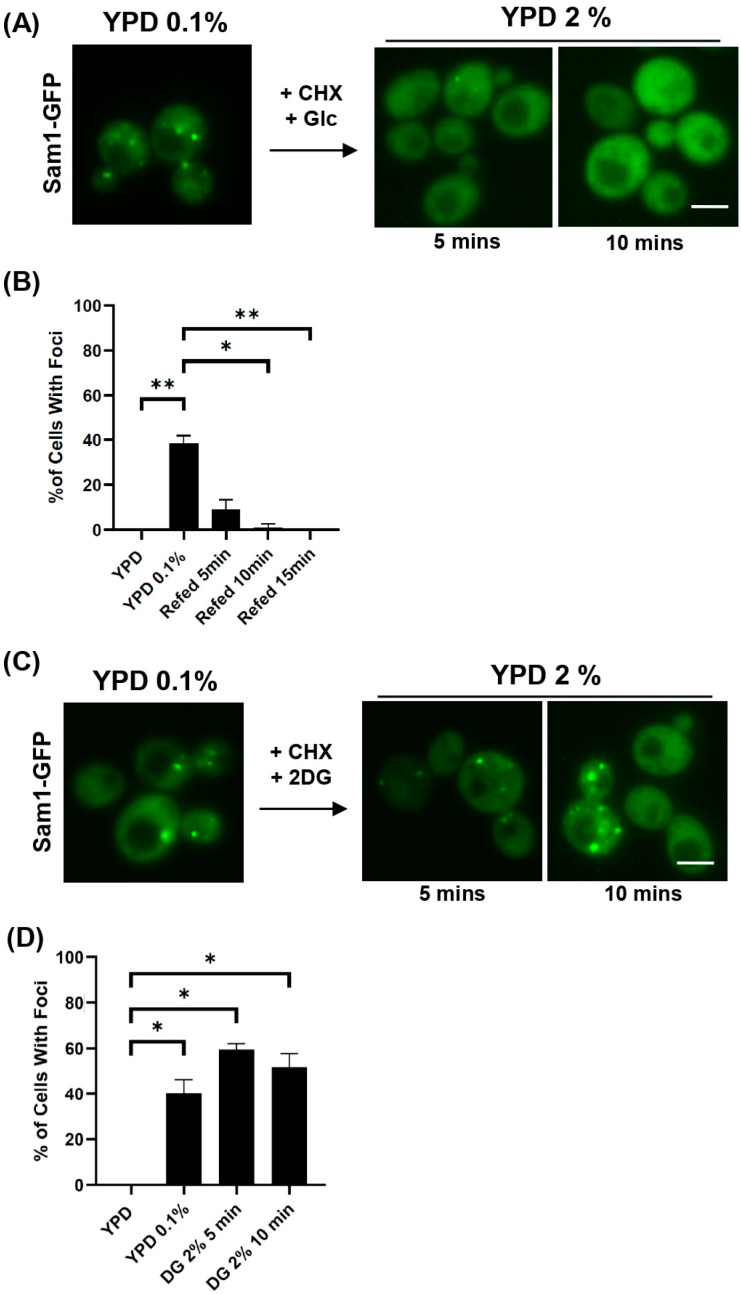
SAM1 assemblies disassemble rapidly in response to metabolizable glucose. (**A**) Cells harboring Sam1-GFP foci after growing in YPD 0.1% for 8 h were transferred to YPD 2% for 5–10 min. Cycloheximide (CHX) was added to block new protein synthesis. Imaging shows rapid Sam1-GFP foci disassembly. (**B**) Quantification of cells with Sam1-GFP foci in (**A**). *n* = 3 independent experiments; *n* = 50 cells per condition. Statistical significance between groups was assessed using an unpaired two-tailed Student’s *t*-test. A *p*-value < 0.05 was considered statistically significant (* *p* < 0.05, ** *p* < 0.01). (**C**) Cells harboring Sam1-GFP foci after growing in YPD 0.1% for 8 h were transferred to YPD 2% with 2-deoxyglucose (2DG), and CHX, a non-metabolizable glucose analog, prevents foci disassembly, indicating a requirement for active glucose metabolism. (**D**) Quantification of cells with Sam1-GFP foci in (**C**). *n* = 3 independent experiments; *n* = 50 cells per condition; * *p* < 0.05, unpaired *t*-test. Scale bars, 2 μm.

To test this, cells were grown under glucose-limited conditions to induce Sam1-GFP foci formation and then subjected to glucose refeeding in the presence of cycloheximide (CHX) to inhibit new protein synthesis. This approach allowed us to specifically monitor the behavior of pre-existing Sam1-GFP assemblies during refeeding, rather than newly synthesized protein arising after glucose addition. Upon glucose addition, Sam1-GFP foci rapidly dissolved within minutes (Figure 3A,B), indicating that Sam1 relocalization is reversible and does not require de novo protein synthesis. These observations support the interpretation that Sam1 assemblies represent dynamic, regulated structures rather than irreversible aggregates.

We next asked whether this dissolution requires glucose metabolism. To address this, we performed similar refeeding experiments in the presence of 2-deoxyglucose (2-DG), a glucose analog that is taken up and phosphorylated but cannot be further metabolized, thereby inhibiting glycolytic flux. Under these conditions, Sam1-GFP foci persisted despite the presence of extracellular glucose (Figure 3C,D). These results demonstrate that disassembly of Sam1 foci requires metabolizable glucose and is therefore dependent on metabolic activity. This finding indicates that Sam1 relocalization is regulated by metabolic flux rather than glucose availability alone.

### 3.4. Sam1 Regulated Assemblies Are Independent of Autophagy and Aggregation Pathways

Cytosolic foci formed under stress can arise from multiple processes, including regulated enzyme assemblies, protein aggregation, or targeting for degradation pathways [30,31]. We therefore asked whether Sam1 foci formation depends on autophagy or canonical aggregation machinery.

**Figure 4:**
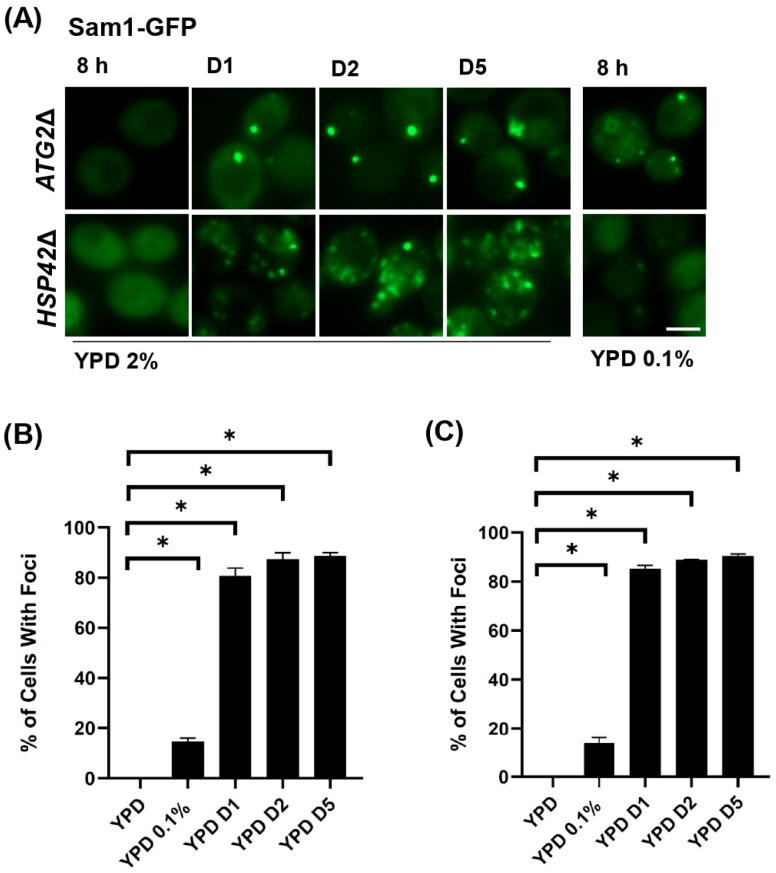
Sam1 foci formation persists in autophagy- and chaperone-deficient cells. (**A**) Representative images of Sam1-GFP localization in *ATG2*Δ and *HSP42*Δ yeast strains during gradual and acute glucose starvation. Cells were grown to log phase in YPD (2% glucose) and starved for the indicated durations (8 h, 1 d, 2 d, and 5 d) or subjected to low-glucose conditions (YPD 0.1%) for 8 h. Sam1-GFP foci are observed in both *ATG2*Δ and *HSP42*Δ strains across all conditions, indicating that foci formation occurs independently of autophagy and chaperone-mediated aggregation pathways. Single-plane images are shown. (**B**) Quantification of the percentage of *ATG2*Δ cells containing Sam1-GFP foci under the indicated starvation conditions. *n* = 3 independent experiments; *n* = 50 cells per condition. Data represent the proportion of total cells exhibiting foci. Student’s *t*-test (* *p* < 0.05). (**C**) Quantification of the percentage of *HSP42*Δ cells containing Sam1-GFP foci under the indicated starvation conditions. Data represent the proportion of total cells exhibiting foci. *n* = 3 independent experiments; *n* = 50 cells per condition, Student’s *t*-test (* *p* < 0.05). Scale bars: 2 μm.

To address this, we examined Sam1-GFP localization in cells lacking *ATG2*, a core component required for autophagosome formation, and *HSP42*, a small heat shock protein associated with protein aggregation and sequestration. In both *ATG2*Δ and *HSP42*Δ backgrounds, Sam1-GFP foci formed at levels comparable to wild-type cells under glucose-limited conditions and during progressive starvation (Figure 4A–C). These findings indicate that Sam1 foci formation occurs independently of autophagy and canonical aggregation pathways, consistent with the interpretation that Sam1 assembles into regulated, reversible structures rather than misfolded protein aggregates or degradation intermediates.

### 3.5. Sam1 Relocalization Occurs Within a Metabolically Rewired State Characterized by Selective Pathway Remodeling

To define the metabolic context associated with Sam1 relocalization, we performed metabolomic profiling of cells grown under glucose-replete (YPD, 2% glucose) and glucose-limited (YPD, 0.1% glucose) conditions, which robustly induce Sam1 foci formation.

**Figure 5:**
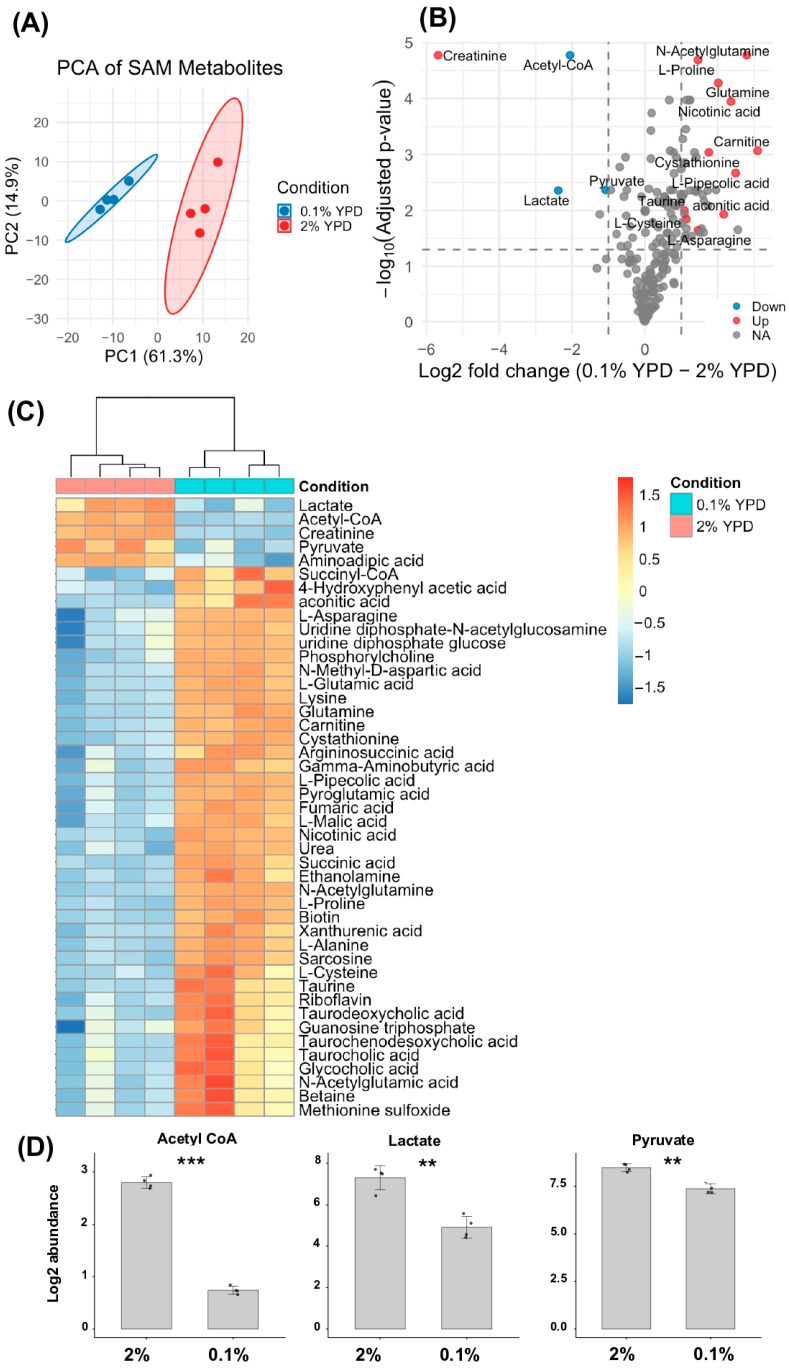
Metabolic remodeling associated with Sam1 relocalization under low-glucose conditions. (**A**) Principal component analysis (PCA) of log_2_-transformed metabolite abundances from cells grown in 0.1% and 2% YPD. Each point represents a biological replicate (*n* = 4); ellipses denote 95% confidence intervals. (**B**) Volcano plot showing log_2_ fold change (0.1% vs. 2% YPD) versus −log_10_ (FDR-adjusted *p*-value). Significant metabolites (FDR < 0.05, |log_2_FC| > 1) are highlighted and labeled. (**C**) Heatmap of significantly altered metabolites (FDR < 0.05, |log_2_FC| > 1), showing coordinated metabolic changes under low-glucose conditions. Data are row-scaled and hierarchically clustered. (**D**) Abundance of representative metabolites from the central carbon metabolic pathway. Bars show mean log_2_ abundance ± SD with individual replicates. Significance: ** *p* < 0.01, *** *p* < 0.001.

Principal component analysis (PCA) revealed clear separation between conditions, indicating that glucose limitation drives a distinct and reproducible metabolic state (Figure 5A). Consistent with this, volcano plot analysis identified a subset of metabolites that were significantly altered under glucose limitation (Figure 5B), and hierarchical clustering further demonstrated coordinated remodeling across metabolic pathways (Figure 5C and Figure S2A).

Examination of significantly altered metabolites revealed a coordinated reduction in glycolytic and fermentative carbon metabolism, including decreased levels of acetyl-CoA, pyruvate, and lactate (Figure 5D), consistent with reduced glucose utilization under low-glucose conditions. In contrast, metabolites associated with amino acid and nitrogen metabolism were broadly elevated, including glutamine, glutamate, proline, lysine, L-pipecolic acid, argininosuccinate, and pyroglutamic acid, indicating substantial remodeling of amino acid utilization pathways during nutrient limitation. In parallel, several mitochondrial and TCA-associated intermediates, including aconitic acid, fumarate, malate, succinate, succinyl-CoA, and carnitine, were elevated, suggesting redistribution of metabolic flux toward mitochondrial and respiratory metabolism.

Low-glucose conditions also induced pronounced changes in sulfur and redox-associated metabolism. Metabolites including cystathionine, taurine, cysteine, methionine sulfoxide, and glutathione-related intermediates increased, consistent with activation of oxidative stress and sulfur metabolic pathways (Figure 5D). Consistent with this metabolic signature, oxidative stress induced by H_2_O_2_ treatment also triggered robust Sam1-GFP relocalization (Figure S2B,C), indicating that Sam1 assembly formation occurs under multiple forms of metabolic stress.

Interestingly, despite these widespread metabolic changes, AdoMet did not emerge among the most strongly altered metabolites in the global metabolomics dataset (Figure S2D), suggesting that bulk AdoMet pools remain relatively buffered under low-glucose conditions.

Together, these data demonstrate that Sam1 relocalization occurs within a coordinated metabolic transition characterized by reduced central carbon metabolism, increased amino acid and sulfur metabolism, and oxidative stress signatures.

### 3.6. Sam1 Relocalization Is Associated with Modest Changes in Cellular AdoMet Levels

**Figure 6:**
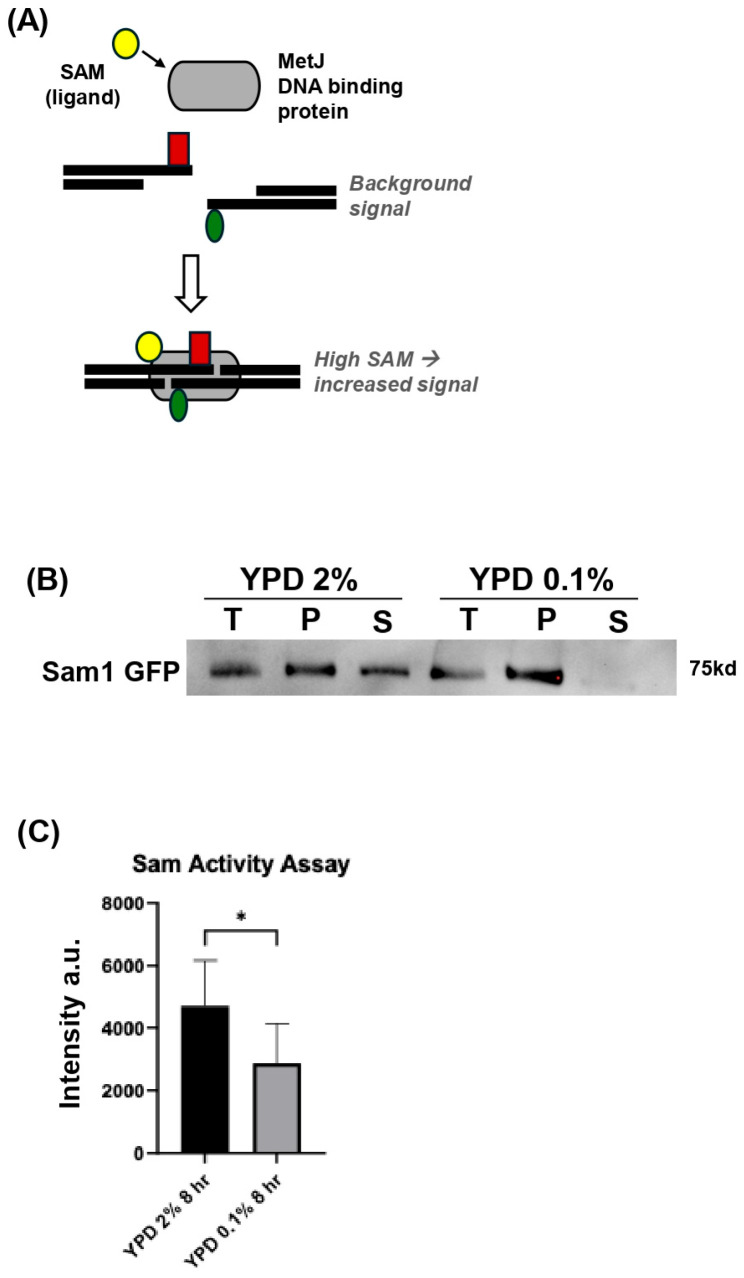
Sam1 relocalization is associated with biochemical partitioning and modest changes in SAM levels. (**A**) Schematic of the Bridge-It^®^ S-Adenosyl Methionine (AdoMet) Fluorescence Assay used to measure total cellular AdoMet. SAM-dependent reactions are coupled to a fluorescence-based readout proportional to AdoMet concentration in yeast lysates. (**B**) Biochemical fractionation of Sam1-GFP under glucose-replete (2% YPD) and glucose-limited (0.1% YPD) conditions. Total (T), pellet (P), and soluble (S) fractions are shown. Under glucose limitation, Sam1 is enriched in the insoluble (pellet) fraction. (**C**) Quantification of total cellular AdoMet levels as fluorescent intensity measured using the Bridge-It^®^ assay under the indicated conditions. Bars represent mean ± SD from *n* = 8 biological replicates. Statistical analyses were conducted on all replicates using the independent samples *t*-test. Normality was assessed with the Shapiro–Wilk test, which showed no significant deviation from a normal distribution. A *p*-value of <0.05 was considered statistically significant (* *p* < 0.05).

Given the central role of Sam1 in AdoMet biosynthesis, we next asked whether targeted measurement of AdoMet levels could detect more subtle changes associated with Sam1 relocalization. To address this, we measured total cellular AdoMet using a Bridge-IT enzymatic assay, in which AdoMet-dependent reactions are coupled to a fluorescence readout proportional to AdoMet concentration (Figure 6A).

Because this assay is performed on cell lysates, we first asked whether Sam1 assemblies persist following cell lysis and could therefore influence the measured AdoMet pool. To test this, we performed biochemical fractionation and Western blotting of Sam1-GFP under glucose-replete and glucose-limited conditions. Under glucose limitation (YPD 0.1%), Sam1-GFP was predominantly recovered in the insoluble pellet (P) fraction, with only a faint signal remaining in the soluble (S) fraction (Figure 6B and Figure S3A), indicating that the assembled state is maintained following cell lysis.

Using this approach, we observed a modest but reproducible reduction in AdoMet levels under glucose-limited conditions (Figure 6C and Figure S3B) indicating that bulk AdoMet abundance remains relatively buffered despite extensive metabolic remodeling and Sam1 relocalization under acute glucose-limitation.

Together, these findings suggest that substantial spatial redistribution of Sam1 can occur with only modest changes in total cellular AdoMet levels, supporting a model in which AdoMet synthase relocalization reflects broader metabolic adaptation rather than large-scale depletion of AdoMet pools.

## 4. Discussion

In this study, we define the nutrient conditions and dynamics that govern the assembly of the yeast AdoMet synthases Sam1. We show that sustained low-glucose conditions drive robust and reversible relocalization of Sam1 into cytosolic assemblies that form progressively during nutrient limitation and rapidly dissolve upon glucose refeeding (Figure 1A–E and Figure 3A,B). These findings place AdoMet synthase within a growing class of metabolic enzymes that dynamically reorganize in response to nutrient stress, supporting the emerging view that spatial enzyme organization represents an additional layer of metabolic regulation beyond transcriptional and enzymatic control.

Previous studies demonstrated that numerous metabolic enzymes reversibly assemble during starvation and metabolic stress, suggesting that enzyme sequestration may help reorganize metabolic pathways during adaptation to changing nutrient conditions [15]. More recent work on lipid biosynthetic enzymes further showed that starvation-induced assemblies can be reversible, independent of classical aggregation pathways, and potentially linked to regulation of metabolic flux rather than irreversible enzyme inactivation [23]. Our findings extend this concept to AdoMet metabolism and indicate that Sam1 assemblies represent regulated adaptive structures rather than nonspecific protein aggregates. Consistent with this interpretation, Sam1 relocalization occurs independently of autophagy and Hsp42-dependent aggregation pathways and remains highly dynamic upon nutrient restoration (Figure 4A–C).

A central finding of this study is that sustained low-glucose conditions induce a Sam1 assembly state that is distinct from previously described acute glucose deprivation responses [26]. This study demonstrated that AdoMet regulates stress granule assembly and that Sam1 can localize to stress granules during prolonged starvation conditions. Importantly, however, Sam1 remained diffuse during acute glucose deprivation despite robust stress granule formation [26]. Our study identifies acute low-glucose metabolic states that strongly promote Sam1 assembly (Figure 1D). Under prolonged starvation, Sam1 assemblies largely overlap with Ded1-positive stress granules, consistent with previous observations (Figure 1A,B). In contrast, under acute low-glucose conditions, Sam1 foci are more abundant than Ded1 puncta and only partially colocalize with Ded1, indicating that Sam1 can enter assembly states that are not fully explained by canonical stress granule association (Figure 2C–E). Together, these findings suggest that Sam1 relocalization is governed by metabolic context, with stress granule recruitment representing one outcome of a broader assembly program rather than the sole identity of these structures.

Our data further demonstrate that Sam1 assembly dynamics are tightly coupled to metabolic flux. Sam1 assemblies rapidly dissolve upon glucose refeeding even in the presence of cycloheximide, indicating that disassembly reflects remodeling of pre-existing structures rather than turnover through newly synthesized protein (Figure 3A,B). In contrast, inhibition of glucose metabolism with 2-deoxyglucose prevented dissolution despite the presence of extracellular glucose (Figure 3C,D). These findings identify metabolizable carbon flux as a key regulator of Sam1 assembly dynamics and suggest that assembly state is linked more directly to cellular metabolic activity than to nutrient sensing alone.

Metabolomic profiling revealed that Sam1 relocalization occurs within a broader metabolically rewired state characterized by reduced glycolytic and fermentative metabolism alongside increased amino acid, sulfur, mitochondrial, and redox-associated pathways (Figure 5B–D). Although untargeted metabolomics indicated that bulk intracellular AdoMet levels remained relatively stable, a targeted fluorescence-based enzymatic assay detected a modest reduction under low-glucose conditions (Figure S2D and Figure 5A–C). These measurements quantify the same metabolite using distinct analytical approaches that differ in sensitivity and dynamic range and together suggest that glucose limitation induces only modest changes in total cellular AdoMet abundance. Importantly, these findings indicate that Sam1 relocalization is not simply a consequence of large-scale depletion of AdoMet pools. Instead, spatial reorganization of Sam1 may occur as part of a coordinated metabolic adaptation program that preserves global AdoMet homeostasis while potentially altering local enzyme accessibility, pathway organization, or compartment-specific metabolite availability.

Although our data demonstrate a strong association between glucose limitation, metabolic remodeling, and Sam1 assembly formation, we cannot exclude contributions from additional physiological changes that accompany prolonged growth under low-glucose conditions. Reduced glucose availability is expected to influence cellular growth rate, extracellular pH, osmolarity, redox balance, and other stress-responsive pathways, any of which could contribute to assembly formation. However, the coordinated metabolic changes observed by means of metabolomic profiling, together with the rapid and reversible reorganization of Sam1 upon changes in glucose availability, support the interpretation that Sam1 assembly formation is closely linked to the cellular metabolic state. Future studies that independently manipulate these physiological parameters will be valuable for defining their relative contributions to Sam1 assembly formation and regulation.

Together, our findings support a model in which sustained low-glucose conditions promote reversible sequestration of AdoMet synthases as part of a broader metabolic adaptation response. In this model, metabolic remodeling drives the formation of multiple Sam1 assembly states, some associated with stress granules and others remaining distinct. More broadly, this work supports the idea that dynamic spatial organization of metabolic enzymes contributes to cellular adaptation during nutrient stress.

Although the present study demonstrates that Sam1 forms reversible assemblies under low-glucose conditions, the effects of this spatial reorganization on methylation-dependent processes and broader metabolic outputs remain to be determined. The present study was conducted in the prototrophic CEN.PK background, which was selected because it is widely used for quantitative metabolic studies and minimizes potential confounding effects associated with auxotrophic nutrient requirements. Future studies examining additional *S. cerevisiae* genetic backgrounds will help determine the extent to which this mechanism is conserved. In addition, future studies will be required to determine whether Sam1 assemblies influence local AdoMet availability, methylation-dependent processes, or metabolic channeling within stress-associated compartments.

## 5. Conclusions

In conclusion, our study demonstrates that the yeast AdoMet synthase Sam1 undergoes dynamic and reversible spatial reorganization in response to sustained low-glucose metabolic states. We show that these assemblies are regulated by metabolizable carbon flux, occur within a broader metabolically rewired state, and exist in multiple organizational forms with distinct relationships to stress granules. Together, these findings support a model in which spatial sequestration of metabolic enzymes represents an adaptive response to nutrient limitation and highlight spatial enzyme organization as an important layer of metabolic regulation during cellular stress adaptation.

## Data Availability

The raw data supporting the conclusions of this article will be made available by the authors upon request.

## Supplementary Materials

The following supporting information can be downloaded at https://www.mdpi.com/article/10.3390/cells15141267/s1. Figure S1: Additional characterization of Sam1 assembly formation under nutrient-limiting conditions. Figure S2: Quantification of intracellular AdoMet levels and supporting analyses. Figure S3: Uncropped immunoblots corresponding to the Western blot analyses shown in the main figures. Table S1: Yeast strains used in this study; Table S2: Primers used in this study.
