# Supplementary material for: Low-Glucose Metabolic Remodeling Drives Reversible Sequestration of Yeast S-Adenosylmethionine (AdoMet) Synthase
Source: Cells. 2026 Jul 14;15(14):1267. doi: 10.3390/cells15141267 (PMC13406880; doi:10.3390/cells15141267)
Supplement: Supplementary file 1 [file cells-15-01267-s001.zip › cells-4358298-supplementary.pdf]

Supplemental material:

Table S1: Yeast strains used in this study

Strain; Genotype	*Origin*
S. cerevisiae: Name = CEN.PK	(Ye et al., 2017)
S. cerevisiae: Name = CEN.PK; Genotype = Sam1-GFP::KanMX	(Ye et al., 2017)
S. cerevisiae: Name = CEN.PK; Genotype = Sam1-GFP::KanMX - Sam2Δ::Hygro	(Ye et al., 2017)
S. cerevisiae: Name = CEN.PK; Genotype = Sam-mCherry::Hygro	(Ye et al., 2017)
S. cerevisiae: Name = CEN.PK; Genotype = Sam1-GFP::KanMX - Ded1-RFP::Hygro	This study
S. cerevisiae: Name = CEN.PK; Genotype = Sam1-GFP::KanMX - Atg2Δ::Hygro	This study
S. cerevisiae: Name = CEN.PK; Genotype = Sam1-GFP::KanMX - Hsp42Δ::Hygro	This study

Table S2: Primers used in this study

**Primers used for this Study**		
Primers	*Sequence*	*Purpose*
Forward *Ded1*	*AGCGATTCCAAGTCTTCTGGCTGGGGTAACAGCGGTGGTTCAAACAACTCTTCTTGGTGGCGGATCCCCGGGTTAATTAA*	C-terminal RFP tagging
Reverse *Ded1*	*AGAAAATATAAGACATGCTAGAGCAGAAAACGAAGAATCCTCACCCTAGTTTGTCTGAAAGAATTCGAGCTCGTTTAAAC*	C-terminal RFP tagging
Forward *ATG2*	AAATTAAGAGGAACCCTTTTTTTTTTTGATTTCGATACACGGATCCCCGGGTTAATTAA	ATG2 Knock-out
Reverse *ATG2*	TATGAATTGAATATATATCAAAAATGTCTGCAAAAATTTGAATTCGAGCTCGTTTAAAC	ATG2 Knock-out

Forward *HSP42*	CATATCCCACACAAATTAAGATCATACCAAG CCGAAGCACGGATCCCCGGGTTAATTAA	HSP42 Knock-out
Reverse *HSP42*	ATATAAATGTATGTATGTGTGTATAAACAGA TACGATATGAATTCGAGCTCGTTTAAAC	HSP42 Knock-out

Figure legends:

Figure S1. (A) Representative images of Sam1-GFP localization under varying pH conditions (pH 4.5, 5.5, 6.5, 7.5, 8.5) in YPD 2%, observed at 4 and 8 hours. No significant foci formation is induced across the pH range. **(B)** Representative images of Sam1-GFP localization in yeast cells grown in YPD 2% under temperature stress: ice shock (cold stress) or 37°C (heat stress) compared to 30°C control, observed at 1 and 3 hours. No foci formation is observed. **(C)** Representative images showing colocalization of Sam1-GFP and Sam2-mCherry in cells starved in YPD 0.1% for 8 h. Merged images indicate that Sam1 and Sam2 localize to the same foci. **(D)** Representative images of Sam1-GFP localization in in Sam2 knock-out grown in YPD 0.1% for 8 h. Scale bars, 2 µm.

Figure S2. (A) Heatmap of all detected metabolites, row-scaled and hierarchically clustered to show global patterns across conditions. **(B)** Representative images of Sam1-GFP localization in yeast cells grown in YPD 2% and treated with 5 mM H_2O_2 for 1 or 3 hours, showing rapid foci formation within 1 hour compared to untreated controls. **(C)** Quantification of cells in (A) with Sam1-GFP foci under oxidative stress conditions (YPD 2% with 5 mM H_2O_2) for 1 or 3 hours, compared to untreated controls (n = 50 cells per condition; *$p < 0.05$, unpaired t-test). **(D)** Metabolite abundance. Bars show mean $\log_2$ abundance ± SD with individual replicates. Significance: *$p < 0.05$, **$p < 0.01$, ***$p < 0.001$. Scale bars, 2 µm.

Figure S3. (A) Full (uncropped) membrane of Western Blot in Fig. 6B. **(B)** Two independent experiments each measuring the total cellular AdoMet levels as fluorescent intensity using the Bridge-IT enzymatic assay under glucose-replete (2% YPD) and glucose-limited (0.1% YPD) conditions. Exp-1 had 3 independent controls and 3 independent test replicates, (*$p<0.04$); Exp-2 had 5 independent controls and 5 independent test replicates, (*$p< 0.04$). Bars show mean ± SD. Statistical significance was determined by Welch's t-test.

Figure S1

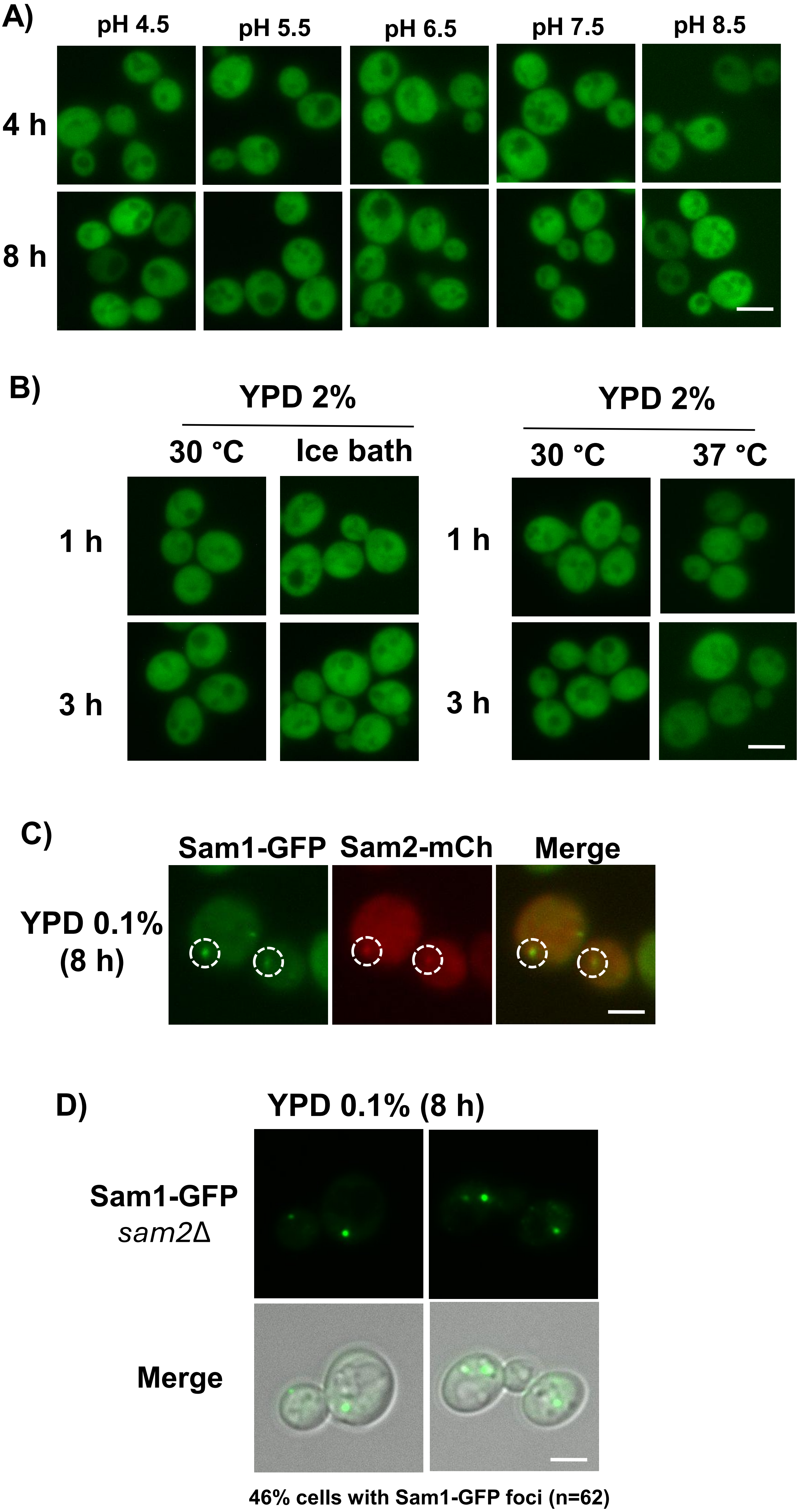
A)
pH 4.5
pH 5.5
pH 6.5
pH 7.5
pH 8.5
4 h
8 h
B)
YPD 2%
30 °C
Ice bath
YPD 2%
30 °C
37 °C
1 h
3 h
1 h
3 h
C)
Sam1-GFP
Sam2-mCh
Merge
YPD 0.1%
(8 h)
D)
YPD 0.1% (8 h)
Sam1-GFP
sam2Δ
Merge
46% cells with Sam1-GFP foci (n=62)

Figure S2

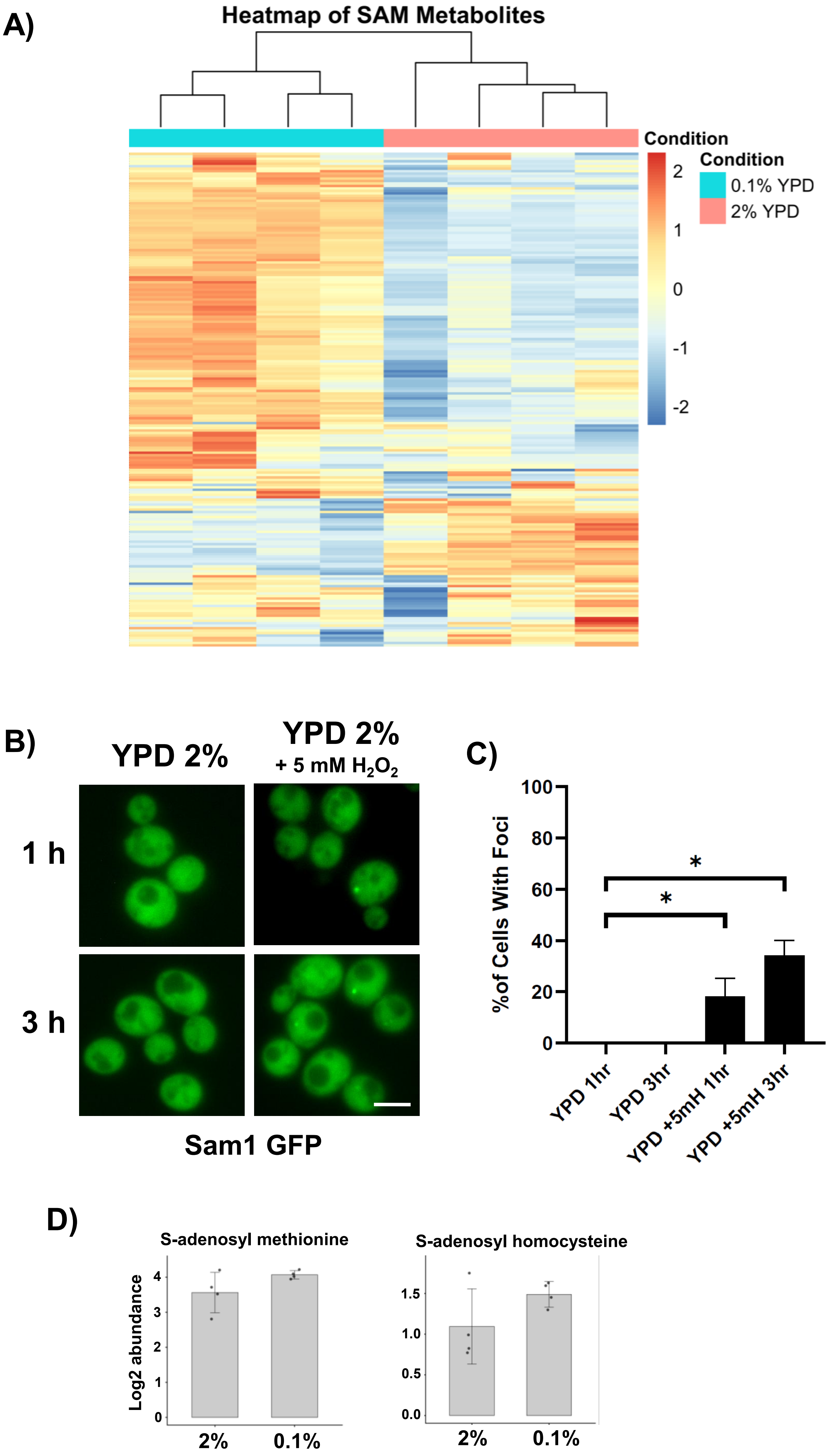
A)
Heatmap of SAM Metabolites
Condition
Condition
0.1% YPD
2% YPD
2
1
0
-1
-2
B)
YPD 2%
YPD 2%
+ 5 mM H_2O_2
1 h
3 h
Sam1 GFP
C)
%of Cells With Foci
100
80
60
40
20
0
*
*
YPD 1hr
YPD 3hr
YPD +5mH 1hr
YPD +5mH 3hr
D)
S-adenosyl methionine
S-adenosyl homocysteine
Log2 abundance
4
3
2
1
0
1.5
1.0
0.5
0.0
2%
0.1%
2%
0.1%

Figure S3

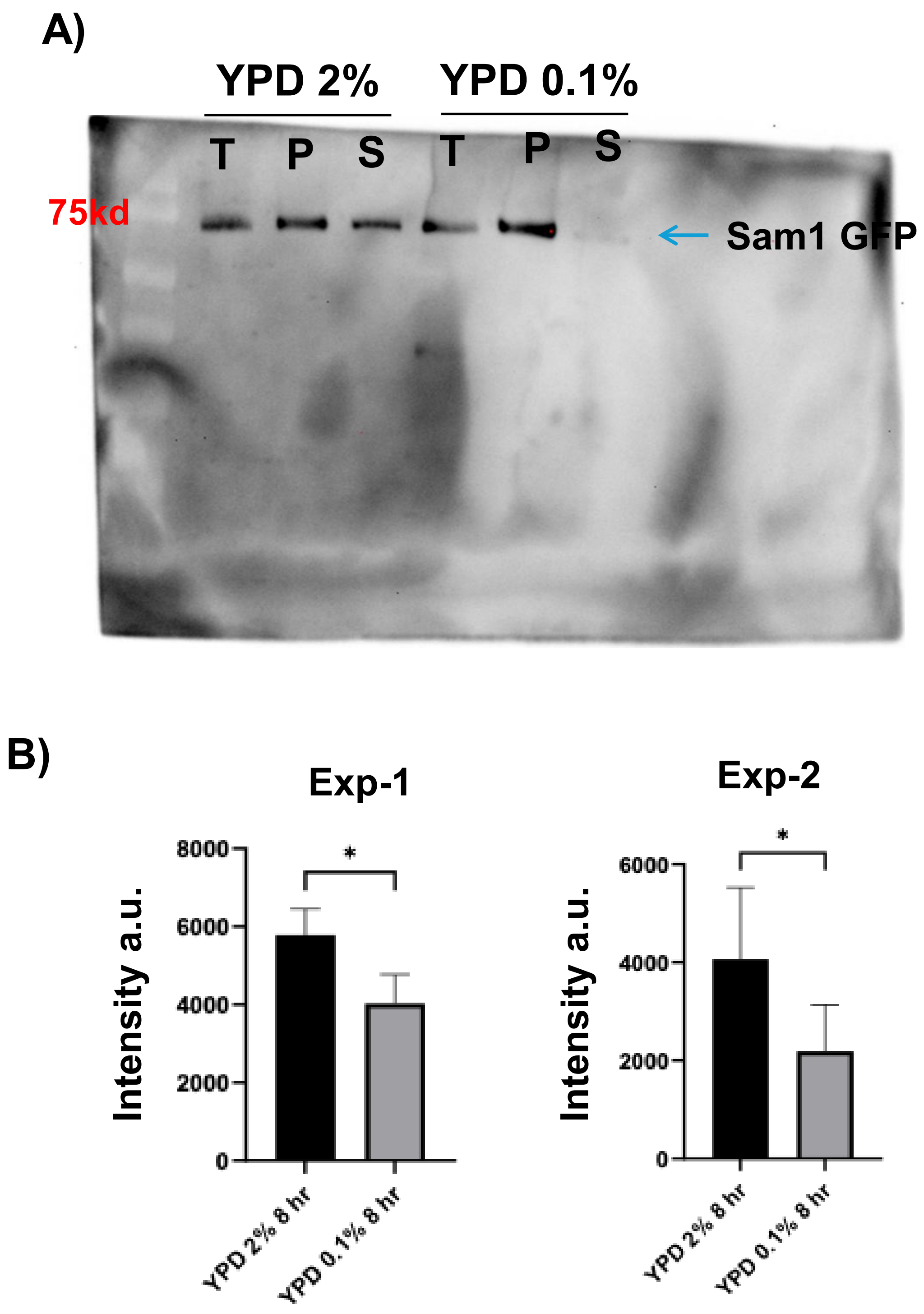
A)
YPD 2%
YPD 0.1%
T P S T P S
75kd
Sam1 GFP
B)
Exp-1
Exp-2
Intensity a.u.
8000
6000
4000
2000
0
*
YPD 2% 8 hr
YPD 0.1% 8 hr
